# 3,5-Lutidine penta­aqua sulfate complexes of first-row transition metals: [*M*(3,5-lutidine)(H_2_O)_5_]SO_4_, with *M* = Mn, Co, Ni, and Zn

**DOI:** 10.1107/S2056989023005261

**Published:** 2023-06-16

**Authors:** James A. Golen, David R. Manke

**Affiliations:** a University of Massachusetts Dartmouth, 285 Old Westport Road, North Dartmouth, MA 02747, USA; University of Durham, United Kingdom

**Keywords:** crystal structure, lutidine, sulfate, transition metal, coordination chemistry

## Abstract

The reactions of MnSO_4_·H_2_O, CoSO_4_·7H_2_O, NiSO_4_·6H_2_O and ZnSO_4_·7H_2_O with 3,5-lutidine (3,5-di­methyl­pyridine) yield crystals of penta­aqua­(3,5-di­methyl­pyridine-κ*N*)manganese(II) sulfate, [Mn(C_7_H_9_N)(H_2_O)_5_]SO_4_, (**1**), penta­aqua­(3,5-di­methyl­pyridine-κ*N*)cobalt(II) sulfate, [Co(C_7_H_9_N)(H_2_O)_5_]SO_4_, (**2**), penta­aqua­(3,5-di­methyl­pyridine-κ*N*)nickel(II) sulfate, [Ni(C_7_H_9_N)(H_2_O)_5_]SO_4_, (**3**), and penta­aqua­(3,5-di­methyl­pyridine-κ*N*)zinc(II) sulfate, [Zn(C_7_H_9_N)(H_2_O)_5_]SO_4_, (**4**), which were characterized by single-crystal X-ray diffraction. The four crystals are isostructural, demonstrating near identical unit-cell parameters and atomic positions. The metal atoms are all octa­hedrally coordinated, with one lutidine ligand and five water ligands. The sulfate dianion hydrogen bonds with the coordinated water mol­ecules of the dicationic metal complex salts, generating infinite three-dimensional networks.

## Abstract 2

The structures of four metal 3,5-lutidine penta­aqua sulfates (Mn, Co, Ni, Zn) are presented and are shown to be isostructural.

## 1. Chemical context

Metal–pyridine sulfate complexes have been reported in the literature since the 1880s (Jørgensen, 1886 ▸; Reitzenstein, 1898 ▸; Manke, 2021 ▸), though an extensive and systematic look at the crystal structures of this class of compounds has never been undertaken. In recent years, our laboratory began looking at the structures of first-row transition-metal–pyridine sulfate complexes, first with the parent pyridine (Park *et al.*, 2019 ▸; Pham *et al.*, 2018 ▸; Roy *et al.*, 2018 ▸) and then with picoline ligands (Park *et al.*, 2022 ▸; Pham *et al.*, 2019 ▸). In our efforts to examine the structural diversity of this class of compounds, we recently expanded to look at lutidine ligands. Herein we report four isostructural first-row transition-metal complexes of 3,5-lutidine. 
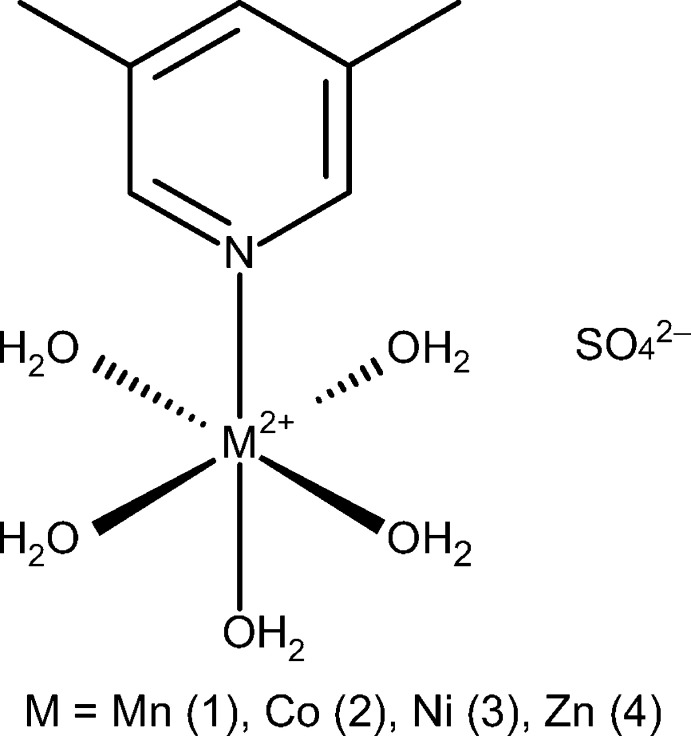


## 2. Structural commentary

**Figure 1:**
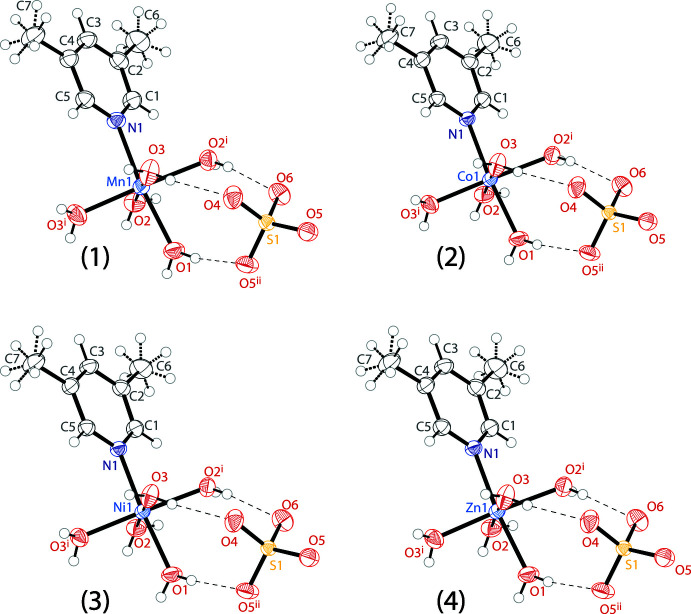
The mol­ecular structures of 3,5-lutidine penta­aqua manganese sulfate (**1**), 3,5-lutidine penta­aqua cobalt sulfate (**2**), 3,5-lutidine penta­aqua nickel sulfate (**3**), and 3,5-lutidine penta­aqua zinc sulfate (**4**) showing the atomic labeling. Displacement ellipsoids are drawn at the 50% probability level. Hydrogen bonds are shown as dashed lines. Dashed bonds are used to show the disordered hydrogen atoms on the methyl groups. Symmetry codes: (i) *x*, 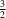 − *y*, *z;* (ii) *x*, 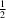 − *y*, *z*.

The four compounds described herein are isostructural, demonstrating near identical unit-cell parameters and atomic positions (Fig. 1 ▸). The asymmetric unit comprises half of the cation and half of the sulfate anion, both ions having crystallographic mirror symmetry. In the cation, the metal atom, the lutidine ligand and the O1 atom of the *trans*-aqua ligand lie in the mirror plane, while two independent aqua ligands are in general positions. In each structure, both methyl groups of the lutidine ligand are rotationally disordered between two mirror-related orientations. In the anion, atoms S1, O4 and O6 lie in the mirror plane, while O5 and O5^ii^ are related by it. Reflection generates the full dicationic complex, which exhibits an octa­hedral coordination with one lutidine and five water ligands bound to the metal, as well as the full sulfate dianion.

The *M*O_3_N plane formed by the three crystallographically unique water mol­ecules and the lutidine nitro­gen atom is rotated by 45.52 (4)° from the plane of the pyridine ring for Mn, 45.79 (4)° for Co, 45.93 (3)° for Ni, and 45.75 (3)° for Zn. The *M*—N distances (Table 1 ▸) observed in the complexes are all consistent with the ionic radii for the metals (Shannon, 1976 ▸). The full sulfate dianions have three near equivalent S—O bonds (S1—O4, S1—O5 and S1—O5^ii^) with two metal-bound waters hydrogen bonding to each oxygen atom. There is also one slightly longer S—O bond (S1—O6) with four metal-bound waters hydrogen bonding to the oxygen. All S—O distances are listed in Table 1 ▸.

## 3. Supra­molecular features

**Figure 2:**
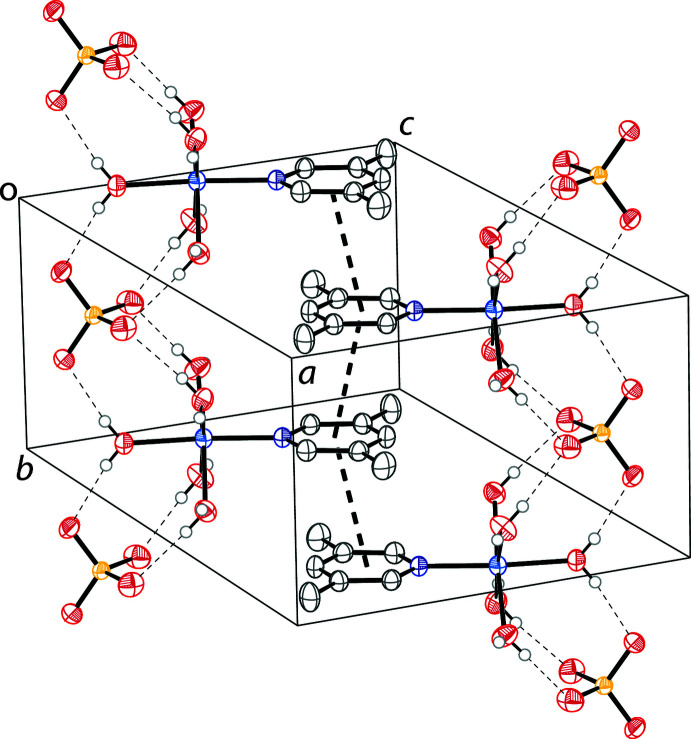
The crystal packing of 3,5-lutidine penta­aqua zinc sulfate (**4**). Displacement ellipsoids are drawn at the 50% probability level. Hydrogen bonds are shown as dashed lines and π–π inter­actions are shown as bold dashed lines. Hydrogen atoms not involved in hydrogen bonding are omitted for clarity.

The ions in all of the compounds described are connected in an extended 3D network through hydrogen bonding. The major hydrogen bonds are between the metal–aqua complexes and the sulfate dianions (Tables 2 ▸–5 ▸ ▸ ▸). The extended structure packing of all compounds show π–π stacking between lutidine rings of adjacent complexes. The parameters of the π–π inter­actions are in Table 6 ▸. The crystal packing of the zinc complex is shown in Fig. 2 ▸. The crystal packing of the other three compounds is isostructural in nature.

## 4. Database survey

While there are many examples of metal–pyridine penta­hydrate complexes, there is only one pyridine-based penta­hydrate complex of a transition metal with a sulfate counter-ion, which is the dimer of zinc bridged by 1,2-bis­(pyridin-3-yl­methyl­ene)hydrazine (YUMVAG; Lozovan *et al.*, 2020 ▸). The other similar structures with sulfur-based anions in the literature include a 3-carboxamide­pyridine complex of cobalt with a sulfonate counter-ion (CACFAP; Lian *et al.*, 2010 ▸), and a pyridine nickel sulfonate complex with a calixarene tetra­sulfonate counter-anion (VIWHUE: Atwood *et al.*, 1991 ▸). The only similar 3,5-lutidine structures are a tetra­kis­(3,5-lutidine) copper sulfate complex (IWAWEJ; Bowmaker *et al.*, 2011 ▸), and a bis­(3,5-lutidine) nickel thio­sulfate dimer (BEMNIS; Pladzyk *et al.*, 2012 ▸).

## 5. Synthesis and crystallization

A metal sulfate (44 mg of MnSO_4_·H_2_O, 44 mg of CoSO_4_·7H_2_O, 217 mg of NiSO_4_·6H_2_O, 33 mg of ZnSO_4_·7H_2_O) was dissolved in five drops of water and 2.5 mL of 3,5-lutidine. The resulting solution was heated to 338–343 K for twelve hours and allowed to cool slowly to room temperature producing single crystals suitable for X-ray diffraction. The manganese crystals formed as colorless blocks, the cobalt crystals formed as pink blocks, the nickel crystals formed as pale-green plates, and the zinc crystals formed as colorless blocks.

## 6. Refinement

Crystal data, data collection and structure refinement details are summarized in Table 7 ▸. The water hydrogen atoms H1, H2*A*, H2*B*, H3*A*, and H3*B* were found in difference-Fourier maps. These hydrogen atoms were refined isotropically, using DFIX restraints with O—H distances of 0.78 (1) Å. Isotopic displacement parameters were set to 1.5 *U*_eq_ of the parent oxygen atom. All other hydrogen atoms were placed in calculated positions [C—H = 0.93 Å (*sp* ^2^), 0.96 Å (CH_3_)]. Isotropic displacement parameters were set to 1.2 *U*_eq_ of the parent aromatic carbon atoms and 1.5 *U*_eq_ of the parent methyl atoms.

## Supplementary Material

Crystal structure: contains datablock(s) 1, 2, 3, 4. DOI: 10.1107/S2056989023005261/zv2026sup1.cif

Structure factors: contains datablock(s) 1. DOI: 10.1107/S2056989023005261/zv20261sup2.hkl

Structure factors: contains datablock(s) 2. DOI: 10.1107/S2056989023005261/zv20262sup3.hkl

Structure factors: contains datablock(s) 3. DOI: 10.1107/S2056989023005261/zv20263sup4.hkl

Structure factors: contains datablock(s) 4. DOI: 10.1107/S2056989023005261/zv20264sup5.hkl

CCDC references: 2269315, 2269314, 2269313, 2269312

Additional supporting information:  crystallographic information; 3D view; checkCIF report

## Figures and Tables

**Table 1:** Selected bond lengths (Å) for compounds (**1**)–(**4**)

Compound	*M*—N1	S1—O4	S1—O5	S1—O6
(**1**)	2.227 (3)	1.462 (2)	1.4650 (17)	1.484 (2)
(**2**)	2.112 (3)	1.462 (3)	1.4618 (17)	1.488 (2)
(**3**)	2.066 (2)	1.464 (2)	1.4588 (14)	1.4895 (19)
(**4**)	2.0924 (19)	1.4641 (19)	1.4596 (13)	1.4886 (18)

**Table 2:** Hydrogen-bond geometry (Å, °) for (**1**)

*D*—H⋯*A*	*D*—H	H⋯*A*	*D*⋯*A*	*D*—H⋯*A*
O1—H1⋯O5^i^	0.79 (1)	2.00 (1)	2.775 (2)	166 (3)
O2—H2*A*⋯O6^i^	0.78 (1)	2.06 (1)	2.832 (3)	172 (3)
O2—H2*B*⋯O6^ii^	0.78 (1)	2.10 (2)	2.850 (3)	162 (4)
O3—H3*A*⋯O5^iii^	0.78 (1)	1.98 (1)	2.752 (2)	177 (4)
O3—H3*B*⋯O4	0.78 (1)	1.99 (1)	2.748 (3)	165 (3)

Symmetry codes: (i) 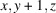 ; (ii) 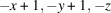 ; (iii) 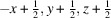 .

**Table 3:** Hydrogen-bond geometry (Å, °) for (**2**)

*D*—H⋯*A*	*D*—H	H⋯*A*	*D*⋯*A*	*D*—H⋯*A*
O1—H1⋯O5^i^	0.78 (1)	2.01 (1)	2.782 (2)	173 (3)
O2—H2*A*⋯O6^i^	0.78 (1)	2.07 (1)	2.840 (3)	169 (3)
O2—H2*B*⋯O6^ii^	0.78 (1)	2.07 (2)	2.822 (3)	161 (4)
O3—H3*A*⋯O5^iii^	0.77 (1)	1.99 (1)	2.764 (2)	174 (3)
O3—H3*B*⋯O4	0.78 (1)	1.97 (1)	2.742 (3)	171 (3)

Symmetry codes: (i) 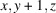 ; (ii) 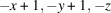 ; (iii) 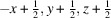 .

**Table 4:** Hydrogen-bond geometry (Å, °) for (**3**)

*D*—H⋯*A*	*D*—H	H⋯*A*	*D*⋯*A*	*D*—H⋯*A*
O1—H1⋯O5^i^	0.78 (1)	2.00 (1)	2.7822 (18)	174 (3)
O2—H2*A*⋯O6^i^	0.78 (1)	2.08 (1)	2.857 (2)	172 (2)
O2—H2*B*⋯O6^ii^	0.79 (1)	2.06 (1)	2.821 (2)	163 (3)
O3—H3*A*⋯O5^iii^	0.77 (1)	2.00 (1)	2.7683 (18)	173 (2)
O3—H3*B*⋯O4	0.77 (1)	1.98 (1)	2.745 (2)	172 (2)

Symmetry codes: (i) 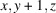 ; (ii) 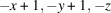 ; (iii) 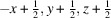 .

**Table 5:** Hydrogen-bond geometry (Å, °) for (**4**)

*D*—H⋯*A*	*D*—H	H⋯*A*	*D*⋯*A*	*D*—H⋯*A*
O1—H1⋯O5^i^	0.78 (1)	2.01 (1)	2.7892 (17)	172 (3)
O2—H2*A*⋯O6^i^	0.78 (1)	2.07 (1)	2.845 (2)	173 (2)
O2—H2*B*⋯O6^ii^	0.79 (1)	2.08 (1)	2.833 (2)	162 (3)
O3—H3*A*⋯O5^iii^	0.77 (1)	1.99 (1)	2.7571 (18)	177 (2)
O3—H3*B*⋯O4	0.77 (1)	1.97 (1)	2.7395 (19)	172 (2)

Symmetry codes: (i) 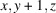 ; (ii) 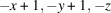 ; (iii) 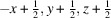 .

**Table 6:** Parameters of π–π inter­actions (Å)

	(**1**)	(**2**)	(**3**)	(**4**)
Centroid-to-centroid	3.6461 (6)	3.6485 (6)	3.6337 (5)	3.6370 (5)
Plane-to-plane shift	0.770 (3)	0.829 (3)	0.8599 (19)	0.8290 (19)
Plane-to-centroid	3.5639 (3)	3.5532 (2)	3.53045 (15)	3.54130 (15)

**Table 7:** Experimental details

	(**1**)	(**2**)	(**3**)	(**4**)
Crystal data				
Chemical formula	[Mn(C_7_H_9_N)(H_2_O)_5_]SO_4_	[Co(C_7_H_9_N)(H_2_O)_5_]SO_4_	[Ni(C_7_H_9_N)(H_2_O)_5_]SO_4_	[Zn(C_7_H_9_N)(H_2_O)_5_]SO_4_
*M*_r_	348.23	352.22	352.00	358.66
Crystal system, space group	Orthorhombic, *P* *n* *m* *a*	Orthorhombic, *P* *n* *m* *a*	Orthorhombic, *P* *n* *m* *a*	Orthorhombic, *P* *n* *m* *a*
Temperature (K)	297	297	297	297
*a*, *b*, *c* (Å)	17.1868 (13), 7.1278 (5), 11.4447 (8)	17.1238 (10), 7.1064 (4), 11.2576 (6)	17.1196 (8), 7.0609 (3), 11.2233 (5)	17.1312 (8), 7.0826 (3), 11.2879 (5)
*V* (Å^3^)	1402.02 (17)	1369.92 (13)	1356.67 (10)	1369.60 (11)
*Z*	4	4	4	4
Radiation type	Mo *K*α	Mo *K*α	Mo *K*α	Mo *K*α
μ (mm^−1^)	1.13	1.44	1.62	1.99
Crystal size (mm)	0.22 × 0.08 × 0.05	0.08 × 0.08 × 0.06	0.17 × 0.04 × 0.03	0.21 × 0.13 × 0.1

Data collection				
Diffractometer	Bruker D8 Venture CMOS	Bruker D8 Venture CMOS	Bruker D8 Venture CMOS	Bruker D8 Venture CMOS
Absorption correction	Multi-scan (*SADABS*; Bruker, 2021 ▸)	Multi-scan (*SADABS*; Bruker, 2021 ▸)	Multi-scan (*SADABS*; Bruker, 2021 ▸)	Multi-scan (*SADABS*; Bruker, 2021 ▸)
*T*_min_, *T*_max_	0.585, 0.745	0.714, 0.745	0.680, 0.745	0.671, 0.745
No. of measured, independent and observed [*I* > 2σ(*I*)] reflections	27161, 1460, 1265	32289, 1367, 1194	36924, 1499, 1386	46710, 1516, 1414
*R*_int_	0.074	0.070	0.049	0.042
(sin θ/λ)_max_ (Å^−1^)	0.612	0.603	0.625	0.625

Refinement				
*R*[*F* ^2^ > 2σ(*F* ^2^)], *wR*(*F* ^2^), *S*	0.032, 0.081, 1.11	0.028, 0.069, 1.12	0.023, 0.060, 1.10	0.021, 0.056, 1.12
No. of reflections	1460	1367	1499	1516
No. of parameters	128	128	128	128
No. of restraints	7	7	7	7
H-atom treatment	H atoms treated by a mixture of independent and constrained refinement	H atoms treated by a mixture of independent and constrained refinement	H atoms treated by a mixture of independent and constrained refinement	H atoms treated by a mixture of independent and constrained refinement
Δρ_max_, Δρ_min_ (e Å^−3^)	0.39, −0.42	0.43, −0.29	0.44, −0.33	0.40, −0.27

Computer programs: *APEX4* and *SAINT* (Bruker, 2021 ▸), *SHELXT2014* (Sheldrick, 2015*a* ▸), *SHELXL2018* (Sheldrick, 2015*b* ▸), *OLEX2* (Dolomanov *et al.*, 2009 ▸), and *publCIF* (Westrip, 2010 ▸).

## supplementary crystallographic information

### Pentaaqua(3,5-dimethylpyridine-κN)manganese(II) sulfate (1) Crystal data

**Table:**

[Mn(C_7_H_9_N)(H_2_O)_5_]SO_4_	*D*_x_ = 1.650 Mg m^−^^3^
*M**_r_* = 348.23	Mo *K*α radiation, λ = 0.71073 Å
Orthorhombic, *P**n**m**a*	Cell parameters from 7487 reflections
*a* = 17.1868 (13) Å	θ = 3.0–25.7°
*b* = 7.1278 (5) Å	µ = 1.13 mm^−^^1^
*c* = 11.4447 (8) Å	*T* = 297 K
*V* = 1402.02 (17) Å^3^	BLOCK, colourless
*Z* = 4	0.22 × 0.08 × 0.05 mm
*F*(000) = 724	

### Pentaaqua(3,5-dimethylpyridine-κN)manganese(II) sulfate (1) Data collection

**Table:**

Bruker D8 Venture CMOS diffractometer	1265 reflections with *I* > 2σ(*I*)
φ and ω scans	*R*_int_ = 0.074
Absorption correction: multi-scan (SADABS; Bruker, 2021)	θ_max_ = 25.8°, θ_min_ = 3.0°
*T*_min_ = 0.585, *T*_max_ = 0.745	*h* = −20→20
27161 measured reflections	*k* = −8→8
1460 independent reflections	*l* = −13→13

### Pentaaqua(3,5-dimethylpyridine-κN)manganese(II) sulfate (1) Refinement

**Table:**

Refinement on *F*^2^	7 restraints
Least-squares matrix: full	Hydrogen site location: mixed
*R*[*F*^2^ > 2σ(*F*^2^)] = 0.032	H atoms treated by a mixture of independent and constrained refinement
*wR*(*F*^2^) = 0.081	*w* = 1/[σ^2^(*F*_o_^2^) + (0.0433*P*)^2^ + 0.6101*P*] where *P* = (*F*_o_^2^ + 2*F*_c_^2^)/3
*S* = 1.11	(Δ/σ)_max_ < 0.001
1460 reflections	Δρ_max_ = 0.39 e Å^−^^3^
128 parameters	Δρ_min_ = −0.42 e Å^−^^3^

### Pentaaqua(3,5-dimethylpyridine-κN)manganese(II) sulfate (1) Special details

**Table:**

Geometry. All esds (except the esd in the dihedral angle between two l.s. planes) are estimated using the full covariance matrix. The cell esds are taken into account individually in the estimation of esds in distances, angles and torsion angles; correlations between esds in cell parameters are only used when they are defined by crystal symmetry. An approximate (isotropic) treatment of cell esds is used for estimating esds involving l.s. planes.

### Pentaaqua(3,5-dimethylpyridine-κN)manganese(II) sulfate (1) Fractional atomic coordinates and isotropic or equivalent isotropic displacement parameters (Å^2^)

**Table:**

	*x*	*y*	*z*	*U*_iso_*/*U*_eq_	Occ. (<1)
Mn1	0.38853 (3)	0.750000	0.19452 (4)	0.02628 (16)	
S1	0.33347 (4)	0.250000	−0.03698 (6)	0.02472 (19)	
O1	0.35349 (17)	0.750000	0.0101 (2)	0.0431 (6)	
O2	0.47201 (10)	0.9730 (3)	0.14197 (15)	0.0370 (4)	
O3	0.30623 (11)	0.5330 (3)	0.23922 (17)	0.0442 (4)	
O4	0.29510 (15)	0.250000	0.0770 (2)	0.0442 (6)	
O5	0.31258 (11)	0.0805 (2)	−0.10225 (15)	0.0433 (4)	
O6	0.41874 (13)	0.250000	−0.0163 (2)	0.0414 (6)	
N1	0.44712 (15)	0.750000	0.3681 (2)	0.0305 (6)	
C1	0.52514 (18)	0.750000	0.3748 (3)	0.0335 (7)	
H1A	0.553273	0.750000	0.305376	0.040*	
C2	0.56607 (18)	0.750000	0.4786 (3)	0.0322 (7)	
C3	0.52253 (19)	0.750000	0.5812 (3)	0.0344 (7)	
H3	0.547891	0.750000	0.652995	0.041*	
C4	0.44230 (19)	0.750000	0.5779 (3)	0.0327 (7)	
C5	0.40755 (19)	0.750000	0.4686 (3)	0.0323 (7)	
H5	0.353492	0.750000	0.465014	0.039*	
C6	0.6535 (2)	0.750000	0.4798 (4)	0.0489 (10)	
H6A	0.671766	0.681677	0.546736	0.073*	0.5
H6B	0.672147	0.876855	0.483598	0.073*	0.5
H6C	0.672591	0.691467	0.409899	0.073*	0.5
C7	0.3933 (2)	0.750000	0.6867 (3)	0.0500 (10)	
H7A	0.420061	0.816577	0.747595	0.075*	0.5
H7B	0.384142	0.623070	0.711140	0.075*	0.5
H7C	0.344518	0.810353	0.670925	0.075*	0.5
H1	0.3430 (17)	0.834 (3)	−0.032 (2)	0.054 (9)*	
H2A	0.4583 (16)	1.057 (3)	0.103 (2)	0.056 (10)*	
H2B	0.5081 (14)	0.930 (5)	0.111 (3)	0.084 (13)*	
H3A	0.2719 (12)	0.549 (4)	0.282 (2)	0.062 (10)*	
H3B	0.2951 (17)	0.451 (3)	0.197 (2)	0.062 (10)*	

### Pentaaqua(3,5-dimethylpyridine-κN)manganese(II) sulfate (1) Atomic displacement parameters (Å^2^)

**Table:**

	*U*^11^	*U*^22^	*U*^33^	*U*^12^	*U*^13^	*U*^23^
Mn1	0.0239 (2)	0.0297 (3)	0.0253 (3)	0.000	−0.00054 (17)	0.000
S1	0.0223 (4)	0.0241 (4)	0.0278 (4)	0.000	−0.0028 (3)	0.000
O1	0.0624 (17)	0.0320 (14)	0.0347 (14)	0.000	−0.0176 (12)	0.000
O2	0.0330 (9)	0.0403 (10)	0.0378 (9)	−0.0031 (8)	0.0016 (7)	0.0063 (8)
O3	0.0413 (10)	0.0442 (11)	0.0469 (10)	−0.0143 (9)	0.0136 (8)	−0.0105 (9)
O4	0.0490 (15)	0.0410 (14)	0.0425 (13)	0.000	0.0151 (11)	0.000
O5	0.0548 (11)	0.0305 (9)	0.0447 (9)	0.0012 (8)	−0.0186 (8)	−0.0054 (7)
O6	0.0218 (11)	0.0466 (15)	0.0558 (15)	0.000	−0.0007 (10)	0.000
N1	0.0274 (13)	0.0352 (15)	0.0290 (13)	0.000	−0.0047 (11)	0.000
C1	0.0293 (16)	0.0395 (19)	0.0315 (16)	0.000	0.0012 (13)	0.000
C2	0.0256 (16)	0.0314 (17)	0.0396 (18)	0.000	−0.0050 (13)	0.000
C3	0.0340 (17)	0.0389 (19)	0.0303 (16)	0.000	−0.0080 (13)	0.000
C4	0.0318 (16)	0.0357 (17)	0.0307 (16)	0.000	−0.0015 (13)	0.000
C5	0.0273 (15)	0.0344 (17)	0.0351 (17)	0.000	−0.0038 (13)	0.000
C6	0.0255 (17)	0.064 (3)	0.057 (2)	0.000	−0.0055 (16)	0.000
C7	0.042 (2)	0.076 (3)	0.0317 (18)	0.000	0.0010 (15)	0.000

### Pentaaqua(3,5-dimethylpyridine-κN)manganese(II) sulfate (1) Geometric parameters (Å, º)

**Table:**

Mn1—O1	2.195 (2)	N1—C5	1.336 (4)
Mn1—O2^i^	2.2240 (17)	C1—H1A	0.9300
Mn1—O2	2.2240 (17)	C1—C2	1.381 (5)
Mn1—O3	2.1575 (17)	C2—C3	1.392 (5)
Mn1—O3^i^	2.1575 (17)	C2—C6	1.504 (4)
Mn1—N1	2.227 (3)	C3—H3	0.9300
S1—O4	1.462 (2)	C3—C4	1.379 (5)
S1—O5^ii^	1.4650 (17)	C4—C5	1.386 (4)
S1—O5	1.4650 (17)	C4—C7	1.503 (5)
S1—O6	1.484 (2)	C5—H5	0.9300
O1—H1	0.786 (10)	C6—H6A	0.9600
O1—H1^i^	0.786 (10)	C6—H6B	0.9600
O2—H2A	0.781 (10)	C6—H6C	0.9600
O2—H2B	0.776 (10)	C7—H7A	0.9600
O3—H3A	0.775 (10)	C7—H7B	0.9600
O3—H3B	0.779 (10)	C7—H7C	0.9600
N1—C1	1.343 (4)		
			
O1—Mn1—O2^i^	85.24 (7)	C1—N1—Mn1	120.2 (2)
O1—Mn1—O2	85.24 (7)	C5—N1—Mn1	122.5 (2)
O1—Mn1—N1	169.05 (11)	C5—N1—C1	117.3 (3)
O2^i^—Mn1—O2	91.24 (10)	N1—C1—H1A	118.0
O2^i^—Mn1—N1	87.11 (7)	N1—C1—C2	123.9 (3)
O2—Mn1—N1	87.11 (7)	C2—C1—H1A	118.0
O3^i^—Mn1—O1	92.76 (8)	C1—C2—C3	116.9 (3)
O3—Mn1—O1	92.76 (8)	C1—C2—C6	121.1 (3)
O3—Mn1—O2	177.99 (7)	C3—C2—C6	122.0 (3)
O3^i^—Mn1—O2	88.55 (7)	C2—C3—H3	119.5
O3—Mn1—O2^i^	88.55 (7)	C4—C3—C2	120.9 (3)
O3^i^—Mn1—O2^i^	177.99 (7)	C4—C3—H3	119.5
O3—Mn1—O3^i^	91.59 (11)	C3—C4—C5	117.1 (3)
O3—Mn1—N1	94.87 (7)	C3—C4—C7	122.5 (3)
O3^i^—Mn1—N1	94.87 (7)	C5—C4—C7	120.4 (3)
O4—S1—O5^ii^	110.15 (9)	N1—C5—C4	123.9 (3)
O4—S1—O5	110.15 (10)	N1—C5—H5	118.1
O4—S1—O6	107.66 (15)	C4—C5—H5	118.1
O5—S1—O5^ii^	111.09 (14)	C2—C6—H6A	109.5
O5—S1—O6	108.85 (9)	C2—C6—H6B	109.5
O5^ii^—S1—O6	108.86 (9)	C2—C6—H6C	109.5
Mn1—O1—H1^i^	130 (2)	H6A—C6—H6B	109.5
Mn1—O1—H1	130 (2)	H6A—C6—H6C	109.5
H1—O1—H1^i^	99 (4)	H6B—C6—H6C	109.5
Mn1—O2—H2A	120 (2)	C4—C7—H7A	109.5
Mn1—O2—H2B	111 (3)	C4—C7—H7B	109.5
H2A—O2—H2B	106.7 (17)	C4—C7—H7C	109.5
Mn1—O3—H3A	123 (2)	H7A—C7—H7B	109.5
Mn1—O3—H3B	123 (2)	H7A—C7—H7C	109.5
H3A—O3—H3B	108.1 (17)	H7B—C7—H7C	109.5
			
Mn1—N1—C1—C2	180.000 (1)	C2—C3—C4—C5	0.000 (1)
Mn1—N1—C5—C4	180.000 (1)	C2—C3—C4—C7	180.000 (1)
N1—C1—C2—C3	0.000 (1)	C3—C4—C5—N1	0.000 (1)
N1—C1—C2—C6	180.000 (1)	C5—N1—C1—C2	0.000 (1)
C1—N1—C5—C4	0.000 (1)	C6—C2—C3—C4	180.000 (1)
C1—C2—C3—C4	0.000 (1)	C7—C4—C5—N1	180.000 (1)

Symmetry codes: (i) *x*, −*y*+3/2, *z*; (ii) *x*, −*y*+1/2, *z*.

### Pentaaqua(3,5-dimethylpyridine-κN)manganese(II) sulfate (1) Hydrogen-bond geometry (Å, º)

**Table:**

*D*—H···*A*	*D*—H	H···*A*	*D*···*A*	*D*—H···*A*
O1—H1···O5^iii^	0.79 (1)	2.00 (1)	2.775 (2)	166 (3)
O2—H2*A*···O6^iii^	0.78 (1)	2.06 (1)	2.832 (3)	172 (3)
O2—H2*B*···O6^iv^	0.78 (1)	2.10 (2)	2.850 (3)	162 (4)
O3—H3*A*···O5^v^	0.78 (1)	1.98 (1)	2.752 (2)	177 (4)
O3—H3*B*···O4	0.78 (1)	1.99 (1)	2.748 (3)	165 (3)

Symmetry codes: (iii) *x*, *y*+1, *z*; (iv) −*x*+1, −*y*+1, −*z*; (v) −*x*+1/2, *y*+1/2, *z*+1/2.

### Pentaaqua(3,5-dimethylpyridine-κN)cobalt(II) sulfate (2) Crystal data

**Table:**

[Co(C_7_H_9_N)(H_2_O)_5_]SO_4_	*D*_x_ = 1.708 Mg m^−^^3^
*M**_r_* = 352.22	Mo *K*α radiation, λ = 0.71073 Å
Orthorhombic, *P**n**m**a*	Cell parameters from 6866 reflections
*a* = 17.1238 (10) Å	θ = 3.0–25.3°
*b* = 7.1064 (4) Å	µ = 1.44 mm^−^^1^
*c* = 11.2576 (6) Å	*T* = 297 K
*V* = 1369.92 (13) Å^3^	BLOCK, pink
*Z* = 4	0.08 × 0.08 × 0.06 mm
*F*(000) = 732	

### Pentaaqua(3,5-dimethylpyridine-κN)cobalt(II) sulfate (2) Data collection

**Table:**

Bruker D8 Venture CMOS diffractometer	1194 reflections with *I* > 2σ(*I*)
φ and ω scans	*R*_int_ = 0.070
Absorption correction: multi-scan (SADABS; Bruker, 2021)	θ_max_ = 25.4°, θ_min_ = 3.0°
*T*_min_ = 0.714, *T*_max_ = 0.745	*h* = −20→20
32289 measured reflections	*k* = −8→8
1367 independent reflections	*l* = −13→13

### Pentaaqua(3,5-dimethylpyridine-κN)cobalt(II) sulfate (2) Refinement

**Table:**

Refinement on *F*^2^	7 restraints
Least-squares matrix: full	Hydrogen site location: mixed
*R*[*F*^2^ > 2σ(*F*^2^)] = 0.028	H atoms treated by a mixture of independent and constrained refinement
*wR*(*F*^2^) = 0.069	*w* = 1/[σ^2^(*F*_o_^2^) + (0.033*P*)^2^ + 0.9563*P*] where *P* = (*F*_o_^2^ + 2*F*_c_^2^)/3
*S* = 1.12	(Δ/σ)_max_ < 0.001
1367 reflections	Δρ_max_ = 0.43 e Å^−^^3^
128 parameters	Δρ_min_ = −0.29 e Å^−^^3^

### Pentaaqua(3,5-dimethylpyridine-κN)cobalt(II) sulfate (2) Special details

**Table:**

Geometry. All esds (except the esd in the dihedral angle between two l.s. planes) are estimated using the full covariance matrix. The cell esds are taken into account individually in the estimation of esds in distances, angles and torsion angles; correlations between esds in cell parameters are only used when they are defined by crystal symmetry. An approximate (isotropic) treatment of cell esds is used for estimating esds involving l.s. planes.

### Pentaaqua(3,5-dimethylpyridine-κN)cobalt(II) sulfate (2) Fractional atomic coordinates and isotropic or equivalent isotropic displacement parameters (Å^2^)

**Table:**

	*x*	*y*	*z*	*U*_iso_*/*U*_eq_	Occ. (<1)
Co1	0.38970 (2)	0.750000	0.19737 (4)	0.02337 (15)	
S1	0.33394 (4)	0.250000	−0.03379 (7)	0.02209 (19)	
O1	0.35093 (18)	0.750000	0.0198 (2)	0.0394 (6)	
O2	0.46967 (11)	0.9655 (3)	0.14383 (16)	0.0336 (4)	
O3	0.31132 (11)	0.5387 (3)	0.24411 (17)	0.0384 (4)	
O4	0.29778 (16)	0.250000	0.0839 (2)	0.0427 (7)	
O5	0.31138 (11)	0.0802 (2)	−0.09856 (16)	0.0406 (5)	
O6	0.42011 (14)	0.250000	−0.0164 (2)	0.0396 (6)	
N1	0.44535 (16)	0.750000	0.3648 (2)	0.0251 (6)	
C1	0.5235 (2)	0.750000	0.3707 (3)	0.0293 (7)	
H1A	0.551507	0.750000	0.299987	0.035*	
C2	0.56474 (19)	0.750000	0.4765 (3)	0.0279 (7)	
C3	0.5217 (2)	0.750000	0.5803 (3)	0.0315 (8)	
H3	0.547380	0.750000	0.653096	0.038*	
C4	0.4411 (2)	0.750000	0.5776 (3)	0.0297 (7)	
C5	0.40575 (19)	0.750000	0.4670 (3)	0.0268 (7)	
H5	0.351489	0.750000	0.463642	0.032*	
C6	0.6528 (2)	0.750000	0.4759 (4)	0.0439 (10)	
H6A	0.671655	0.688585	0.546196	0.066*	0.5
H6B	0.671452	0.877337	0.474120	0.066*	0.5
H6C	0.671263	0.684078	0.406972	0.066*	0.5
C7	0.3926 (2)	0.750000	0.6890 (3)	0.0465 (10)	
H7A	0.420270	0.814262	0.751028	0.070*	0.5
H7B	0.382469	0.622638	0.712901	0.070*	0.5
H7C	0.343972	0.813100	0.674071	0.070*	0.5
H1	0.3369 (18)	0.838 (3)	−0.016 (2)	0.058 (10)*	
H2A	0.4543 (17)	1.052 (3)	0.108 (2)	0.058 (11)*	
H2B	0.5060 (14)	0.928 (5)	0.108 (3)	0.079 (14)*	
H3A	0.2753 (11)	0.555 (4)	0.284 (2)	0.046 (9)*	
H3B	0.3023 (16)	0.457 (3)	0.201 (2)	0.053 (10)*	

### Pentaaqua(3,5-dimethylpyridine-κN)cobalt(II) sulfate (2) Atomic displacement parameters (Å^2^)

**Table:**

	*U*^11^	*U*^22^	*U*^33^	*U*^12^	*U*^13^	*U*^23^
Co1	0.0234 (2)	0.0267 (2)	0.0200 (2)	0.000	−0.00065 (17)	0.000
S1	0.0216 (4)	0.0213 (4)	0.0233 (4)	0.000	−0.0020 (3)	0.000
O1	0.0592 (18)	0.0293 (15)	0.0298 (14)	0.000	−0.0181 (13)	0.000
O2	0.0318 (10)	0.0375 (10)	0.0316 (9)	−0.0022 (8)	0.0004 (8)	0.0067 (9)
O3	0.0381 (11)	0.0389 (11)	0.0382 (10)	−0.0127 (9)	0.0118 (9)	−0.0095 (9)
O4	0.0531 (17)	0.0397 (15)	0.0354 (14)	0.000	0.0166 (12)	0.000
O5	0.0531 (11)	0.0261 (10)	0.0427 (10)	−0.0013 (8)	−0.0190 (9)	−0.0058 (8)
O6	0.0228 (12)	0.0426 (15)	0.0532 (16)	0.000	−0.0015 (11)	0.000
N1	0.0255 (14)	0.0280 (15)	0.0218 (13)	0.000	−0.0028 (11)	0.000
C1	0.0298 (18)	0.0311 (18)	0.0270 (17)	0.000	0.0007 (14)	0.000
C2	0.0270 (17)	0.0258 (17)	0.0309 (18)	0.000	−0.0047 (14)	0.000
C3	0.0344 (19)	0.036 (2)	0.0245 (16)	0.000	−0.0085 (14)	0.000
C4	0.0341 (18)	0.0292 (18)	0.0258 (17)	0.000	0.0003 (14)	0.000
C5	0.0248 (16)	0.0290 (17)	0.0266 (17)	0.000	−0.0031 (13)	0.000
C6	0.0238 (19)	0.060 (3)	0.048 (2)	0.000	−0.0031 (16)	0.000
C7	0.043 (2)	0.070 (3)	0.0268 (19)	0.000	0.0016 (17)	0.000

### Pentaaqua(3,5-dimethylpyridine-κN)cobalt(II) sulfate (2) Geometric parameters (Å, º)

**Table:**

Co1—O1	2.106 (3)	N1—C5	1.335 (4)
Co1—O2	2.1408 (18)	C1—H1A	0.9300
Co1—O2^i^	2.1408 (18)	C1—C2	1.384 (5)
Co1—O3	2.0813 (18)	C2—C3	1.382 (5)
Co1—O3^i^	2.0813 (18)	C2—C6	1.507 (5)
Co1—N1	2.112 (3)	C3—H3	0.9300
S1—O4	1.462 (3)	C3—C4	1.380 (5)
S1—O5^ii^	1.4618 (17)	C4—C5	1.385 (5)
S1—O5	1.4618 (17)	C4—C7	1.504 (5)
S1—O6	1.488 (2)	C5—H5	0.9300
O1—H1^i^	0.778 (10)	C6—H6A	0.9600
O1—H1	0.778 (10)	C6—H6B	0.9600
O2—H2A	0.782 (10)	C6—H6C	0.9600
O2—H2B	0.784 (10)	C7—H7A	0.9600
O3—H3A	0.774 (10)	C7—H7B	0.9600
O3—H3B	0.776 (10)	C7—H7C	0.9600
N1—C1	1.339 (4)		
			
O1—Co1—O2^i^	86.24 (8)	C1—N1—Co1	119.7 (2)
O1—Co1—O2	86.24 (8)	C5—N1—Co1	122.7 (2)
O1—Co1—N1	171.55 (11)	C5—N1—C1	117.7 (3)
O2—Co1—O2^i^	91.32 (11)	N1—C1—H1A	118.2
O3—Co1—O1	92.10 (8)	N1—C1—C2	123.6 (3)
O3^i^—Co1—O1	92.10 (8)	C2—C1—H1A	118.2
O3^i^—Co1—O2	88.15 (8)	C1—C2—C6	120.5 (3)
O3—Co1—O2^i^	88.15 (8)	C3—C2—C1	117.1 (3)
O3^i^—Co1—O2^i^	178.29 (8)	C3—C2—C6	122.5 (3)
O3—Co1—O2	178.29 (8)	C2—C3—H3	119.5
O3—Co1—O3^i^	92.32 (11)	C4—C3—C2	121.0 (3)
O3^i^—Co1—N1	93.74 (8)	C4—C3—H3	119.5
O3—Co1—N1	93.74 (8)	C3—C4—C5	117.2 (3)
N1—Co1—O2	87.86 (7)	C3—C4—C7	122.3 (3)
N1—Co1—O2^i^	87.86 (7)	C5—C4—C7	120.5 (3)
O4—S1—O6	107.51 (16)	N1—C5—C4	123.6 (3)
O5^ii^—S1—O4	109.87 (10)	N1—C5—H5	118.2
O5—S1—O4	109.87 (10)	C4—C5—H5	118.2
O5^ii^—S1—O5	111.27 (14)	C2—C6—H6A	109.5
O5^ii^—S1—O6	109.12 (10)	C2—C6—H6B	109.5
O5—S1—O6	109.12 (10)	C2—C6—H6C	109.5
Co1—O1—H1	126 (2)	H6A—C6—H6B	109.5
Co1—O1—H1^i^	126 (2)	H6A—C6—H6C	109.5
H1—O1—H1^i^	106 (5)	H6B—C6—H6C	109.5
Co1—O2—H2A	120 (2)	C4—C7—H7A	109.5
Co1—O2—H2B	114 (3)	C4—C7—H7B	109.5
H2A—O2—H2B	105.7 (16)	C4—C7—H7C	109.5
Co1—O3—H3A	124 (2)	H7A—C7—H7B	109.5
Co1—O3—H3B	121 (2)	H7A—C7—H7C	109.5
H3A—O3—H3B	108.7 (17)	H7B—C7—H7C	109.5
			
Co1—N1—C1—C2	180.000 (1)	C2—C3—C4—C5	0.000 (1)
Co1—N1—C5—C4	180.000 (1)	C2—C3—C4—C7	180.000 (1)
N1—C1—C2—C3	0.000 (1)	C3—C4—C5—N1	0.000 (1)
N1—C1—C2—C6	180.000 (1)	C5—N1—C1—C2	0.000 (1)
C1—N1—C5—C4	0.000 (1)	C6—C2—C3—C4	180.000 (1)
C1—C2—C3—C4	0.000 (1)	C7—C4—C5—N1	180.000 (1)

Symmetry codes: (i) *x*, −*y*+3/2, *z*; (ii) *x*, −*y*+1/2, *z*.

### Pentaaqua(3,5-dimethylpyridine-κN)cobalt(II) sulfate (2) Hydrogen-bond geometry (Å, º)

**Table:**

*D*—H···*A*	*D*—H	H···*A*	*D*···*A*	*D*—H···*A*
O1—H1···O5^iii^	0.78 (1)	2.01 (1)	2.782 (2)	173 (3)
O2—H2*A*···O6^iii^	0.78 (1)	2.07 (1)	2.840 (3)	169 (3)
O2—H2*B*···O6^iv^	0.78 (1)	2.07 (2)	2.822 (3)	161 (4)
O3—H3*A*···O5^v^	0.77 (1)	1.99 (1)	2.764 (2)	174 (3)
O3—H3*B*···O4	0.78 (1)	1.97 (1)	2.742 (3)	171 (3)

Symmetry codes: (iii) *x*, *y*+1, *z*; (iv) −*x*+1, −*y*+1, −*z*; (v) −*x*+1/2, *y*+1/2, *z*+1/2.

### Pentaaqua(3,5-dimethylpyridine-κN)nickel(II) sulfate (3) Crystal data

**Table:**

[Ni(C_7_H_9_N)(H_2_O)_5_]SO_4_	*D*_x_ = 1.723 Mg m^−^^3^
*M**_r_* = 352.00	Mo *K*α radiation, λ = 0.71073 Å
Orthorhombic, *P**n**m**a*	Cell parameters from 9899 reflections
*a* = 17.1196 (8) Å	θ = 3.0–26.3°
*b* = 7.0609 (3) Å	µ = 1.62 mm^−^^1^
*c* = 11.2233 (5) Å	*T* = 297 K
*V* = 1356.67 (10) Å^3^	BLOCK, green
*Z* = 4	0.17 × 0.04 × 0.03 mm
*F*(000) = 736	

### Pentaaqua(3,5-dimethylpyridine-κN)nickel(II) sulfate (3) Data collection

**Table:**

Bruker D8 Venture CMOS diffractometer	1386 reflections with *I* > 2σ(*I*)
φ and ω scans	*R*_int_ = 0.049
Absorption correction: multi-scan (SADABS; Bruker, 2021)	θ_max_ = 26.4°, θ_min_ = 3.6°
*T*_min_ = 0.680, *T*_max_ = 0.745	*h* = −21→20
36924 measured reflections	*k* = −8→8
1499 independent reflections	*l* = −14→14

### Pentaaqua(3,5-dimethylpyridine-κN)nickel(II) sulfate (3) Refinement

**Table:**

Refinement on *F*^2^	7 restraints
Least-squares matrix: full	Hydrogen site location: mixed
*R*[*F*^2^ > 2σ(*F*^2^)] = 0.023	H atoms treated by a mixture of independent and constrained refinement
*wR*(*F*^2^) = 0.060	*w* = 1/[σ^2^(*F*_o_^2^) + (0.0308*P*)^2^ + 0.6821*P*] where *P* = (*F*_o_^2^ + 2*F*_c_^2^)/3
*S* = 1.10	(Δ/σ)_max_ < 0.001
1499 reflections	Δρ_max_ = 0.44 e Å^−^^3^
128 parameters	Δρ_min_ = −0.33 e Å^−^^3^

### Pentaaqua(3,5-dimethylpyridine-κN)nickel(II) sulfate (3) Special details

**Table:**

Geometry. All esds (except the esd in the dihedral angle between two l.s. planes) are estimated using the full covariance matrix. The cell esds are taken into account individually in the estimation of esds in distances, angles and torsion angles; correlations between esds in cell parameters are only used when they are defined by crystal symmetry. An approximate (isotropic) treatment of cell esds is used for estimating esds involving l.s. planes.

### Pentaaqua(3,5-dimethylpyridine-κN)nickel(II) sulfate (3) Fractional atomic coordinates and isotropic or equivalent isotropic displacement parameters (Å^2^)

**Table:**

	*x*	*y*	*z*	*U*_iso_*/*U*_eq_	Occ. (<1)
Ni1	0.39091 (2)	0.750000	0.19852 (3)	0.02146 (11)	
S1	0.33343 (3)	0.250000	−0.03397 (5)	0.02160 (14)	
O1	0.35007 (14)	0.750000	0.02346 (18)	0.0384 (5)	
O2	0.46878 (7)	0.9627 (2)	0.14536 (12)	0.0309 (3)	
O3	0.31406 (8)	0.5401 (2)	0.24601 (12)	0.0354 (3)	
O4	0.29830 (12)	0.250000	0.08496 (18)	0.0424 (5)	
O5	0.31029 (8)	0.0794 (2)	−0.09827 (12)	0.0410 (3)	
O6	0.41976 (10)	0.250000	−0.01750 (19)	0.0394 (5)	
N1	0.44466 (12)	0.750000	0.36335 (17)	0.0241 (4)	
C1	0.52308 (14)	0.750000	0.3692 (2)	0.0277 (5)	
H1A	0.551105	0.750000	0.298181	0.033*	
C2	0.56433 (14)	0.750000	0.4753 (2)	0.0272 (5)	
C3	0.52130 (15)	0.750000	0.5799 (2)	0.0304 (6)	
H3	0.546992	0.750000	0.652878	0.036*	
C4	0.44033 (15)	0.750000	0.5768 (2)	0.0282 (5)	
C5	0.40464 (14)	0.750000	0.4658 (2)	0.0260 (5)	
H5	0.350357	0.750000	0.462450	0.031*	
C6	0.65243 (15)	0.750000	0.4748 (3)	0.0409 (7)	
H6A	0.671277	0.686857	0.544900	0.061*	0.5
H6B	0.671120	0.878180	0.474031	0.061*	0.5
H6C	0.670966	0.684963	0.405226	0.061*	0.5
C7	0.39189 (17)	0.750000	0.6886 (2)	0.0429 (7)	
H7A	0.418880	0.818472	0.750001	0.064*	0.5
H7B	0.383346	0.621918	0.714204	0.064*	0.5
H7C	0.342529	0.809610	0.673085	0.064*	0.5
H1	0.3354 (14)	0.840 (3)	−0.011 (2)	0.057 (8)*	
H2A	0.4540 (12)	1.048 (2)	0.1067 (18)	0.045 (7)*	
H2B	0.5056 (10)	0.923 (3)	0.1115 (19)	0.062 (9)*	
H3A	0.2777 (9)	0.557 (3)	0.2854 (16)	0.041 (6)*	
H3B	0.3050 (12)	0.460 (3)	0.2012 (16)	0.048 (7)*	

### Pentaaqua(3,5-dimethylpyridine-κN)nickel(II) sulfate (3) Atomic displacement parameters (Å^2^)

**Table:**

	*U*^11^	*U*^22^	*U*^33^	*U*^12^	*U*^13^	*U*^23^
Ni1	0.02095 (16)	0.02450 (18)	0.01894 (17)	0.000	−0.00045 (11)	0.000
S1	0.0213 (3)	0.0206 (3)	0.0229 (3)	0.000	−0.0023 (2)	0.000
O1	0.0564 (13)	0.0289 (11)	0.0300 (10)	0.000	−0.0187 (9)	0.000
O2	0.0301 (7)	0.0325 (7)	0.0300 (7)	−0.0018 (6)	0.0016 (5)	0.0060 (6)
O3	0.0348 (7)	0.0370 (8)	0.0343 (7)	−0.0105 (6)	0.0108 (6)	−0.0078 (7)
O4	0.0527 (12)	0.0386 (12)	0.0357 (11)	0.000	0.0161 (9)	0.000
O5	0.0544 (8)	0.0269 (7)	0.0417 (8)	0.0008 (6)	−0.0201 (6)	−0.0060 (6)
O6	0.0232 (9)	0.0435 (12)	0.0515 (12)	0.000	0.0003 (8)	0.000
N1	0.0243 (9)	0.0260 (11)	0.0221 (10)	0.000	−0.0029 (8)	0.000
C1	0.0252 (11)	0.0302 (14)	0.0277 (13)	0.000	0.0008 (10)	0.000
C2	0.0244 (12)	0.0273 (13)	0.0299 (13)	0.000	−0.0044 (10)	0.000
C3	0.0330 (13)	0.0339 (14)	0.0242 (12)	0.000	−0.0086 (10)	0.000
C4	0.0323 (12)	0.0280 (13)	0.0243 (12)	0.000	0.0004 (10)	0.000
C5	0.0251 (11)	0.0271 (13)	0.0258 (12)	0.000	−0.0022 (9)	0.000
C6	0.0219 (12)	0.0562 (19)	0.0444 (17)	0.000	−0.0065 (11)	0.000
C7	0.0392 (15)	0.064 (2)	0.0251 (14)	0.000	0.0012 (11)	0.000

### Pentaaqua(3,5-dimethylpyridine-κN)nickel(II) sulfate (3) Geometric parameters (Å, º)

**Table:**

Ni1—O1	2.0855 (19)	N1—C5	1.339 (3)
Ni1—O2	2.0949 (13)	C1—H1A	0.9300
Ni1—O2^i^	2.0949 (13)	C1—C2	1.384 (3)
Ni1—O3	2.0522 (13)	C2—C3	1.386 (4)
Ni1—O3^i^	2.0522 (13)	C2—C6	1.508 (3)
Ni1—N1	2.066 (2)	C3—H3	0.9300
S1—O4	1.464 (2)	C3—C4	1.387 (4)
S1—O5^ii^	1.4588 (14)	C4—C5	1.387 (3)
S1—O5	1.4588 (14)	C4—C7	1.505 (4)
S1—O6	1.4895 (19)	C5—H5	0.9300
O1—H1^i^	0.782 (10)	C6—H6A	0.9600
O1—H1	0.782 (10)	C6—H6B	0.9600
O2—H2A	0.782 (9)	C6—H6C	0.9600
O2—H2B	0.787 (9)	C7—H7A	0.9600
O3—H3A	0.773 (9)	C7—H7B	0.9600
O3—H3B	0.774 (9)	C7—H7C	0.9600
N1—C1	1.344 (3)		
			
O1—Ni1—O2^i^	86.85 (6)	C1—N1—Ni1	119.23 (17)
O1—Ni1—O2	86.85 (6)	C5—N1—Ni1	122.77 (16)
O2^i^—Ni1—O2	91.60 (8)	C5—N1—C1	118.0 (2)
O3^i^—Ni1—O1	91.70 (6)	N1—C1—H1A	118.3
O3—Ni1—O1	91.70 (6)	N1—C1—C2	123.4 (2)
O3^i^—Ni1—O2	87.95 (6)	C2—C1—H1A	118.3
O3—Ni1—O2	178.51 (6)	C1—C2—C3	117.2 (2)
O3—Ni1—O2^i^	87.95 (6)	C1—C2—C6	120.5 (2)
O3^i^—Ni1—O2^i^	178.51 (6)	C3—C2—C6	122.3 (2)
O3—Ni1—O3^i^	92.46 (8)	C2—C3—H3	119.7
O3^i^—Ni1—N1	93.04 (6)	C2—C3—C4	120.7 (2)
O3—Ni1—N1	93.04 (6)	C4—C3—H3	119.7
N1—Ni1—O1	173.14 (9)	C3—C4—C5	117.6 (2)
N1—Ni1—O2	88.38 (5)	C3—C4—C7	122.0 (2)
N1—Ni1—O2^i^	88.37 (5)	C5—C4—C7	120.4 (2)
O4—S1—O6	107.12 (13)	N1—C5—C4	123.1 (2)
O5^ii^—S1—O4	109.84 (8)	N1—C5—H5	118.5
O5—S1—O4	109.85 (8)	C4—C5—H5	118.5
O5^ii^—S1—O5	111.30 (11)	C2—C6—H6A	109.5
O5—S1—O6	109.32 (8)	C2—C6—H6B	109.5
O5^ii^—S1—O6	109.32 (8)	C2—C6—H6C	109.5
Ni1—O1—H1	124.8 (19)	H6A—C6—H6B	109.5
Ni1—O1—H1^i^	124.8 (19)	H6A—C6—H6C	109.5
H1—O1—H1^i^	108 (4)	H6B—C6—H6C	109.5
Ni1—O2—H2A	120.2 (17)	C4—C7—H7A	109.5
Ni1—O2—H2B	113.1 (19)	C4—C7—H7B	109.5
H2A—O2—H2B	105.3 (15)	C4—C7—H7C	109.5
Ni1—O3—H3A	123.6 (16)	H7A—C7—H7B	109.5
Ni1—O3—H3B	119.3 (16)	H7A—C7—H7C	109.5
H3A—O3—H3B	108.9 (15)	H7B—C7—H7C	109.5
			
Ni1—N1—C1—C2	180.000 (1)	C2—C3—C4—C5	0.000 (1)
Ni1—N1—C5—C4	180.000 (1)	C2—C3—C4—C7	180.000 (1)
N1—C1—C2—C3	0.000 (1)	C3—C4—C5—N1	0.000 (1)
N1—C1—C2—C6	180.000 (1)	C5—N1—C1—C2	0.000 (1)
C1—N1—C5—C4	0.000 (1)	C6—C2—C3—C4	180.000 (1)
C1—C2—C3—C4	0.000 (1)	C7—C4—C5—N1	180.000 (1)

Symmetry codes: (i) *x*, −*y*+3/2, *z*; (ii) *x*, −*y*+1/2, *z*.

### Pentaaqua(3,5-dimethylpyridine-κN)nickel(II) sulfate (3) Hydrogen-bond geometry (Å, º)

**Table:**

*D*—H···*A*	*D*—H	H···*A*	*D*···*A*	*D*—H···*A*
O1—H1···O5^iii^	0.78 (1)	2.00 (1)	2.7822 (18)	174 (3)
O2—H2*A*···O6^iii^	0.78 (1)	2.08 (1)	2.857 (2)	172 (2)
O2—H2*B*···O6^iv^	0.79 (1)	2.06 (1)	2.821 (2)	163 (3)
O3—H3*A*···O5^v^	0.77 (1)	2.00 (1)	2.7683 (18)	173 (2)
O3—H3*B*···O4	0.77 (1)	1.98 (1)	2.745 (2)	172 (2)

Symmetry codes: (iii) *x*, *y*+1, *z*; (iv) −*x*+1, −*y*+1, −*z*; (v) −*x*+1/2, *y*+1/2, *z*+1/2.

### Pentaaqua(3,5-dimethylpyridine-κN)zinc(II) sulfate (4) Crystal data

**Table:**

[Zn(C_7_H_9_N)(H_2_O)_5_]SO_4_	*D*_x_ = 1.739 Mg m^−^^3^
*M**_r_* = 358.66	Mo *K*α radiation, λ = 0.71073 Å
Orthorhombic, *P**n**m**a*	Cell parameters from 9907 reflections
*a* = 17.1312 (8) Å	θ = 3.0–26.1°
*b* = 7.0826 (3) Å	µ = 1.99 mm^−^^1^
*c* = 11.2879 (5) Å	*T* = 297 K
*V* = 1369.60 (11) Å^3^	BLOCK, colourless
*Z* = 4	0.21 × 0.13 × 0.1 mm
*F*(000) = 744	

### Pentaaqua(3,5-dimethylpyridine-κN)zinc(II) sulfate (4) Data collection

**Table:**

Bruker D8 Venture CMOS diffractometer	1414 reflections with *I* > 2σ(*I*)
φ and ω scans	*R*_int_ = 0.042
Absorption correction: multi-scan (SADABS; Bruker, 2021)	θ_max_ = 26.4°, θ_min_ = 3.0°
*T*_min_ = 0.671, *T*_max_ = 0.745	*h* = −21→21
46710 measured reflections	*k* = −8→8
1516 independent reflections	*l* = −14→14

### Pentaaqua(3,5-dimethylpyridine-κN)zinc(II) sulfate (4) Refinement

**Table:**

Refinement on *F*^2^	7 restraints
Least-squares matrix: full	Hydrogen site location: mixed
*R*[*F*^2^ > 2σ(*F*^2^)] = 0.021	H atoms treated by a mixture of independent and constrained refinement
*wR*(*F*^2^) = 0.056	*w* = 1/[σ^2^(*F*_o_^2^) + (0.0287*P*)^2^ + 0.6476*P*] where *P* = (*F*_o_^2^ + 2*F*_c_^2^)/3
*S* = 1.12	(Δ/σ)_max_ < 0.001
1516 reflections	Δρ_max_ = 0.40 e Å^−^^3^
128 parameters	Δρ_min_ = −0.27 e Å^−^^3^

### Pentaaqua(3,5-dimethylpyridine-κN)zinc(II) sulfate (4) Special details

**Table:**

Geometry. All esds (except the esd in the dihedral angle between two l.s. planes) are estimated using the full covariance matrix. The cell esds are taken into account individually in the estimation of esds in distances, angles and torsion angles; correlations between esds in cell parameters are only used when they are defined by crystal symmetry. An approximate (isotropic) treatment of cell esds is used for estimating esds involving l.s. planes.

### Pentaaqua(3,5-dimethylpyridine-κN)zinc(II) sulfate (4) Fractional atomic coordinates and isotropic or equivalent isotropic displacement parameters (Å^2^)

**Table:**

	*x*	*y*	*z*	*U*_iso_*/*U*_eq_	Occ. (<1)
Zn1	0.38941 (2)	0.750000	0.20029 (2)	0.02445 (10)	
S1	0.33349 (3)	0.250000	−0.03485 (5)	0.02238 (13)	
O1	0.35283 (14)	0.750000	0.02014 (17)	0.0411 (5)	
O2	0.46998 (8)	0.9672 (2)	0.14481 (11)	0.0334 (3)	
O3	0.31084 (8)	0.5381 (2)	0.24347 (13)	0.0393 (3)	
O4	0.29751 (12)	0.250000	0.08280 (17)	0.0435 (5)	
O5	0.31108 (8)	0.07969 (19)	−0.09923 (12)	0.0418 (3)	
O6	0.41959 (10)	0.250000	−0.01709 (19)	0.0403 (5)	
N1	0.44544 (12)	0.750000	0.36501 (17)	0.0258 (4)	
C1	0.52356 (14)	0.750000	0.3709 (2)	0.0292 (5)	
H1A	0.551519	0.750000	0.300258	0.035*	
C2	0.56501 (14)	0.750000	0.4762 (2)	0.0296 (5)	
C3	0.52173 (15)	0.750000	0.5806 (2)	0.0314 (5)	
H3	0.547367	0.750000	0.653242	0.038*	
C4	0.44114 (15)	0.750000	0.5775 (2)	0.0290 (5)	
C5	0.40543 (14)	0.750000	0.4668 (2)	0.0273 (5)	
H5	0.351191	0.750000	0.463327	0.033*	
C6	0.65285 (15)	0.750000	0.4765 (3)	0.0440 (7)	
H6A	0.671449	0.681907	0.544387	0.066*	0.5
H6B	0.671527	0.877698	0.479610	0.066*	0.5
H6C	0.671616	0.690396	0.405608	0.066*	0.5
C7	0.39253 (17)	0.750000	0.6883 (2)	0.0438 (7)	
H7A	0.419605	0.817081	0.749703	0.066*	0.5
H7B	0.383455	0.622262	0.713159	0.066*	0.5
H7C	0.343476	0.810656	0.672744	0.066*	0.5
H1	0.3379 (13)	0.836 (2)	−0.0171 (18)	0.052 (7)*	
H2A	0.4540 (12)	1.049 (3)	0.1049 (18)	0.050 (7)*	
H2B	0.5065 (11)	0.928 (4)	0.1108 (19)	0.068 (9)*	
H3A	0.2758 (10)	0.550 (3)	0.2857 (16)	0.050 (7)*	
H3B	0.3024 (13)	0.458 (3)	0.1986 (16)	0.049 (7)*	

### Pentaaqua(3,5-dimethylpyridine-κN)zinc(II) sulfate (4) Atomic displacement parameters (Å^2^)

**Table:**

	*U*^11^	*U*^22^	*U*^33^	*U*^12^	*U*^13^	*U*^23^
Zn1	0.02442 (15)	0.02788 (16)	0.02103 (15)	0.000	−0.00072 (10)	0.000
S1	0.0220 (3)	0.0216 (3)	0.0235 (3)	0.000	−0.0025 (2)	0.000
O1	0.0616 (13)	0.0301 (11)	0.0317 (10)	0.000	−0.0188 (9)	0.000
O2	0.0320 (7)	0.0367 (7)	0.0314 (6)	−0.0012 (6)	0.0013 (5)	0.0063 (6)
O3	0.0387 (7)	0.0406 (8)	0.0387 (7)	−0.0129 (6)	0.0121 (6)	−0.0103 (7)
O4	0.0544 (12)	0.0396 (11)	0.0366 (10)	0.000	0.0167 (9)	0.000
O5	0.0541 (8)	0.0274 (7)	0.0438 (7)	0.0002 (6)	−0.0201 (6)	−0.0064 (6)
O6	0.0229 (9)	0.0446 (11)	0.0533 (12)	0.000	0.0002 (8)	0.000
N1	0.0275 (10)	0.0274 (10)	0.0226 (9)	0.000	−0.0034 (8)	0.000
C1	0.0259 (12)	0.0339 (13)	0.0278 (12)	0.000	0.0004 (9)	0.000
C2	0.0265 (12)	0.0284 (12)	0.0340 (13)	0.000	−0.0046 (10)	0.000
C3	0.0331 (13)	0.0351 (14)	0.0259 (12)	0.000	−0.0085 (10)	0.000
C4	0.0318 (12)	0.0310 (13)	0.0242 (11)	0.000	0.0001 (10)	0.000
C5	0.0237 (11)	0.0304 (13)	0.0277 (12)	0.000	−0.0018 (9)	0.000
C6	0.0236 (13)	0.0580 (19)	0.0504 (17)	0.000	−0.0049 (11)	0.000
C7	0.0399 (15)	0.066 (2)	0.0251 (13)	0.000	0.0028 (11)	0.000

### Pentaaqua(3,5-dimethylpyridine-κN)zinc(II) sulfate (4) Geometric parameters (Å, º)

**Table:**

Zn1—O1	2.1279 (19)	N1—C5	1.337 (3)
Zn1—O2	2.1598 (13)	C1—H1A	0.9300
Zn1—O2^i^	2.1598 (13)	C1—C2	1.385 (3)
Zn1—O3	2.0742 (13)	C2—C3	1.392 (4)
Zn1—O3^i^	2.0742 (13)	C2—C6	1.505 (3)
Zn1—N1	2.0924 (19)	C3—H3	0.9300
S1—O4	1.4641 (19)	C3—C4	1.381 (4)
S1—O5^ii^	1.4596 (13)	C4—C5	1.392 (3)
S1—O5	1.4596 (13)	C4—C7	1.502 (3)
S1—O6	1.4886 (18)	C5—H5	0.9300
O1—H1^i^	0.784 (10)	C6—H6A	0.9600
O1—H1	0.784 (10)	C6—H6B	0.9600
O2—H2A	0.784 (9)	C6—H6C	0.9600
O2—H2B	0.785 (9)	C7—H7A	0.9600
O3—H3A	0.771 (9)	C7—H7B	0.9600
O3—H3B	0.773 (9)	C7—H7C	0.9600
N1—C1	1.340 (3)		
			
O1—Zn1—O2^i^	84.90 (6)	C1—N1—Zn1	120.14 (16)
O1—Zn1—O2	84.90 (6)	C5—N1—Zn1	121.87 (16)
O2^i^—Zn1—O2	90.87 (8)	C5—N1—C1	118.0 (2)
O3^i^—Zn1—O1	91.92 (6)	N1—C1—H1A	118.2
O3—Zn1—O1	91.92 (6)	N1—C1—C2	123.7 (2)
O3^i^—Zn1—O2	88.13 (6)	C2—C1—H1A	118.2
O3—Zn1—O2	176.74 (5)	C1—C2—C3	117.0 (2)
O3—Zn1—O2^i^	88.13 (6)	C1—C2—C6	120.9 (2)
O3^i^—Zn1—O2^i^	176.74 (5)	C3—C2—C6	122.1 (2)
O3—Zn1—O3^i^	92.71 (8)	C2—C3—H3	119.6
O3^i^—Zn1—N1	95.10 (6)	C4—C3—C2	120.7 (2)
O3—Zn1—N1	95.10 (6)	C4—C3—H3	119.6
N1—Zn1—O1	169.82 (9)	C3—C4—C5	117.5 (2)
N1—Zn1—O2	87.97 (5)	C3—C4—C7	122.2 (2)
N1—Zn1—O2^i^	87.97 (5)	C5—C4—C7	120.3 (2)
O4—S1—O6	107.16 (12)	N1—C5—C4	123.1 (2)
O5^ii^—S1—O4	109.93 (8)	N1—C5—H5	118.4
O5—S1—O4	109.93 (8)	C4—C5—H5	118.4
O5^ii^—S1—O5	111.46 (11)	C2—C6—H6A	109.5
O5—S1—O6	109.12 (7)	C2—C6—H6B	109.5
O5^ii^—S1—O6	109.12 (7)	C2—C6—H6C	109.5
Zn1—O1—H1	127.5 (18)	H6A—C6—H6B	109.5
Zn1—O1—H1^i^	127.5 (18)	H6A—C6—H6C	109.5
H1—O1—H1^i^	102 (4)	H6B—C6—H6C	109.5
Zn1—O2—H2A	118.1 (17)	C4—C7—H7A	109.5
Zn1—O2—H2B	114 (2)	C4—C7—H7B	109.5
H2A—O2—H2B	105.0 (14)	C4—C7—H7C	109.5
Zn1—O3—H3A	125.1 (17)	H7A—C7—H7B	109.5
Zn1—O3—H3B	119.8 (16)	H7A—C7—H7C	109.5
H3A—O3—H3B	109.6 (16)	H7B—C7—H7C	109.5
			
Zn1—N1—C1—C2	180.000 (1)	C2—C3—C4—C5	0.000 (1)
Zn1—N1—C5—C4	180.000 (1)	C2—C3—C4—C7	180.000 (1)
N1—C1—C2—C3	0.000 (1)	C3—C4—C5—N1	0.000 (1)
N1—C1—C2—C6	180.000 (1)	C5—N1—C1—C2	0.000 (1)
C1—N1—C5—C4	0.000 (1)	C6—C2—C3—C4	180.000 (1)
C1—C2—C3—C4	0.000 (1)	C7—C4—C5—N1	180.000 (1)

Symmetry codes: (i) *x*, −*y*+3/2, *z*; (ii) *x*, −*y*+1/2, *z*.

### Pentaaqua(3,5-dimethylpyridine-κN)zinc(II) sulfate (4) Hydrogen-bond geometry (Å, º)

**Table:**

*D*—H···*A*	*D*—H	H···*A*	*D*···*A*	*D*—H···*A*
O1—H1···O5^iii^	0.78 (1)	2.01 (1)	2.7892 (17)	172 (3)
O2—H2*A*···O6^iii^	0.78 (1)	2.07 (1)	2.845 (2)	173 (2)
O2—H2*B*···O6^iv^	0.79 (1)	2.08 (1)	2.833 (2)	162 (3)
O3—H3*A*···O5^v^	0.77 (1)	1.99 (1)	2.7571 (18)	177 (2)
O3—H3*B*···O4	0.77 (1)	1.97 (1)	2.7395 (19)	172 (2)

Symmetry codes: (iii) *x*, *y*+1, *z*; (iv) −*x*+1, −*y*+1, −*z*; (v) −*x*+1/2, *y*+1/2, *z*+1/2.

## References

[bb1] Atwood, J. L., Orr, G. W., Hamada, F., Vincent, R. L., Bott, S. G. & Robinson, K. D. (1991). *J. Am. Chem. Soc.* **113**, 2760–2761.

[bb2] Bowmaker, G. A., Di Nicola, C., Marchetti, F., Pettinari, C., Skelton, B. W., Somers, N. & White, A. H. (2011). *Inorg. Chim. Acta*, **375**, 31–40.

[bb3] Bruker (2021). *APEX4*, *SAINT*, and *SADABS*. Bruker AXS Inc., Madison, Wisconsin, USA.

[bb4] Dolomanov, O. V., Bourhis, L. J., Gildea, R. J., Howard, J. A. K. & Puschmann, H. (2009). *J. Appl. Cryst.* **42**, 339–341.

[bb5] Jørgensen, S. M. (1886). *J. Prakt. Chem.* **33**, 489–538.

[bb6] Lian, Z., Zhao, N. & Liu, P. (2010). *Z. Kristallogr. New Cryst. Struct.* **225**, 371–373.

[bb7] Lozovan, V., Kravtsov, V. C., Coropceanu, E. B., Siminel, A. V., Kulikova, O. V., Costriucova, N. V. & Fonari, M. S. (2020). *J. Solid State Chem.* **286**, 121312. 10.3390/molecules25235616 PMC7730819 33260394

[bb8] Manke, D. R. (2021). *Bull. Hist. Chem.* **46**, 179–185.

[bb9] Park, A. M., Golen, J. A. & Manke, D. R. (2022). *Acta Cryst.* E**78**, 108–110. 10.1107/S2056989022000780 PMC8819428 35145733

[bb10] Park, A. M., Pham, D. N. K., Golen, J. A. & Manke, D. R. (2019). *Acta Cryst.* E**75**, 1888–1891. 10.1107/S205698901901538X PMC6895941 31871752

[bb11] Pham, D. N. K., Roy, M., Kreider-Mueller, A., Golen, J. A. & Manke, D. R. (2018). *Acta Cryst.* E**74**, 857–861. 10.1107/S2056989018007557 PMC6002833 29951245

[bb12] Pham, D. N. K., Roy, M., Kreider-Mueller, A., Golen, J. A. & Manke, D. R. (2019). *Acta Cryst.* C**75**, 568–574. 10.1107/S2053229619004625 31062714

[bb13] Pladzyk, A., Daca, N. & Ponikiewski, L. (2012). *Z. Anorg. Allg. Chem.* **638**, 1497–1500.

[bb14] Reitzenstein, F. (1898). *Z. Anorg. Chem.* **18**, 253–304.

[bb15] Roy, M., Pham, D. N. K., Kreider-Mueller, A., Golen, J. A. & Manke, D. R. (2018). *Acta Cryst.* C**74**, 263–268. 10.1107/S2053229618001547 29504552

[bb16] Shannon, R. D. (1976). *Acta Cryst.* A**32**, 751–767.

[bb17] Sheldrick, G. M. (2015*a*). *Acta Cryst.* A**71**, 3–8.

[bb18] Sheldrick, G. M. (2015*b*). *Acta Cryst.* C**71**, 3–8.

[bb19] Westrip, S. P. (2010). *J. Appl. Cryst.* **43**, 920–925.

